# Contraceptive Needs Among Women Recently Incarcerated at a Rural Appalachian Jail

**DOI:** 10.1089/whr.2021.0033

**Published:** 2021-07-16

**Authors:** Sophie G. Wenzel, Barbie Zabielski, Shelby Borowski

**Affiliations:** ^1^ Department of Population Health Sciences, Virginia-Maryland College of Veterinary Medicine, Virginia Tech, Blacksburg, Virginia, USA.; ^2^ New River Health District, Virginia Department of Health, Christiansburg, Virginia, USA.; ^3^ Department of Human Development and Family Science, College of Liberal Arts and Human Sciences, Virginia Tech, Blacksburg, Virginia, USA.

**Keywords:** incarceration, jail, contraception, reproductive health, emergency contraception, rural

## Abstract

***Background:*** Incarceration is associated with negative sexual and reproductive health outcomes. We examined contraceptive needs among women incarcerated at a rural Appalachian jail with emphasis on pregnancy history, recent contraceptive use, and current and near-future contraceptive needs.

***Materials and Methods:*** A survey was administered to newly incarcerated women at a jail in Southwest Virginia. It included questions about (1) prior pregnancies; (2) pregnancy intentions, contraceptive use, and sexual activity in the 3 months before jail; (3) unprotected sex in the 5 days before jail; (4) interest in contraceptive education and access during incarceration; and (5) post-release sexual activity, pregnancy, and contraceptive plans.

***Results:*** One hundred ninety-three women completed surveys. Analyses focused on the 95 at risk for pregnancy. Fifty-eight percent of prior pregnancies on which women provided intention information were unintended, with 74% of respondents reporting at least 1 such pregnancy. Ninety-four percent of women reported vaginal intercourse during the 3 months before jail. Only 46% of those who did not want to get pregnant reported consistent contraceptive use. Condoms and withdrawal were the most common methods used. Forty percent of women were eligible for emergency contraception (EC). Most (78%) participants anticipated sex with a man within 6 months of release, and most (63%) did not want to become pregnant within a year of release. Almost half (47%) expressed interest in receiving birth control while in jail.

***Conclusions:*** Results support the need to offer women EC on incarceration, family planning education during confinement, and effective birth control before release.

## Introduction

Justice-involved women face inadequate access to health care before, during, and after incarceration,^1–3^ and incarceration is associated with risky sexual behavior and poor sexual and reproductive health outcomes. Among the reproductive-aged women surveyed at Adult Correctional Institute in Rhode Island who reported not wanting to become pregnant, only 28% consistently used birth control in the 3 months before incarceration, and nearly 84% reported at least 1 previous unintended pregnancy.^4^ Twenty-nine percent of women surveyed on intake at San Francisco County Jail had unprotected vaginal intercourse in the 5 days before jail.^5^ At Cook County Jail in Chicago, almost half of the surveyed women at risk for unintended pregnancy reported unprotected vaginal intercourse during the 5 days prior to incarceration.^6^

The American Public Health Association, World Health Organization, National Commission on Correctional Health Care, and American College of Obstetrics and Gynecology have all published policy statements or committee opinions asserting that incarcerated women should be provided with contraceptive counseling and voluntary access to contraceptives prior to release.^7–10^ The latter two organizations explicitly endorse access to emergency contraception (EC) for incarcerated women,^9,10^ which can prevent pregnancy if received within 5 days after unprotected sex. Nevertheless, only slightly more than a third of United States-based correctional facilities reports offering any contraceptives, and only 4% offer EC.^11^

The current study was conducted at New River Valley Regional Jail (NRVRJ) located in Dublin, Virginia, a Health Resources and Services Administration-designated rural area.^12^ NRVRJ houses male and female adults arrested in the following areas of Southwest Virginia and sentenced to serve up to 1 year or held pending bond or trial: Pulaski, Floyd, Giles, Bland, Wythe, Carroll, and Grayson Counties, and the cities of Radford and Galax. The Appalachian Regional Commission defines all 9 of these geographic areas as Appalachian.^13^ From April 1, 2018, through March 31, 2019, there were a total of 1867 female incarcerations at NRVRJ averaging 26 days in duration.

The Appalachian Region comprises 420 United States counties, spans 13 states from northern Mississippi to southern New York, and is divided into 5 subregions. Appalachia suffers from striking disparities in income, employment, education, and health relative to the rest of the country, with subregional and rural–urban disparities existing within the larger area. Average household income is 20% lower in Appalachia than the national average, is 72% higher in non-Appalachian than Appalachian Virginia, and is 7% and 11% higher, respectively, in Northern and Southern Appalachia than in South Central Appalachia where this study took place.^14^ Cancer, chronic obstructive pulmonary disease, diabetes, heart disease, injury, and stroke mortality rates are all at least 10% higher in Appalachia than nationally, with Central Appalachia experiencing the worst outcomes.^15^ Mortality related to drug and alcohol overdose, suicide, and alcoholic liver disease—collectively termed “diseases of despair”—is 36% higher in the Appalachian Region than in the non-Appalachian United States, and opioid overdose mortality is 46% higher.^16^

With the exception of a 2020 study on birth control use among women recruited from rural Appalachian jails who had been using drugs prior to confinement and a 2020 study that examined condom use among drug-using rural Appalachian women recruited from jails,^17,18^ published research on contraceptive needs among incarcerated women in the United States has been conducted in non-Appalachian urban and metropolitan settings.^19^ Research on contraceptive needs among incarcerated women in rural Appalachia is lacking. This is important, because rural and Appalachian women are at increased risk for poor reproductive health outcomes compared with urban and non-Appalachian women, including unintended pregnancies, low birthweight and premature deliveries, and neonatal abstinence syndrome-affected births (infant withdrawal usually due to prenatal opioid use).^20–23^

Incarceration presents a unique opportunity to engage with a high-risk, underserved population and provide them with education and family planning services, including contraceptive methods to use after release. Our purpose was to assess the sexual health risk profile of women newly incarcerated at NRVRJ, with emphasis on history of unintended pregnancies and unprotected sex and current and near-future contraceptive needs. We examined pregnancy histories, unmet need for contraception during the 3 months before participants' most recent incarcerations, potential need for EC at the time of incarceration, interest in contraceptive education and access during confinement, and likely need for contraception on release.

## Materials and Methods

### Procedures

The subject pool consisted of reproductive-aged women between 18 and 49 years old who were newly incarcerated at NRVRJ, able to read and write in English, and able to complete the informed consent process. Women were deemed ineligible for this study if they were not able to read and write in English or were older than 49 years of age.

The survey used for this study was administered during classification assessment, performed by a trained member of NRVRJ's staff within 72 hours of an inmate's arrival. The classification assessment is performed primarily to determine the most appropriate location within the jail to house an individual based on various factors, including criminal history, current charges, prior history with other inmates, and correctional system history. Women were excluded from the study if determined by correctional staff to be ineligible for the jail's standard classification assessment because they lacked the cognitive ability to understand the process and respond to the questions in a meaningful way (*e.g.*, severely developmentally disabled, suffering from a severe traumatic brain injury, acutely psychotic, undergoing acute drug withdrawal).

Data were collected over a 12-month period between April 2018 and April 2019, during which 415 women were invited by NRVRJ staff to complete an anonymous survey. After the classification assessment, a jail staff member handed the recruitment script, consent information, and survey to a potential participant. Consent was implied in the form of a completed questionnaire. After completing the survey, each participant placed it into a numbered envelope and placed the envelope into a locked box.

Because the study population was particularly vulnerable, special care was taken to ensure that research was conducted in a noncoercive manner. The recruitment script and consent form were written to be culturally appropriate, readable, and clearly communicate that participation was voluntary and would not affect status or treatment in jail. Women were provided with 30 minutes of privacy during which to complete the survey or not and place it into an envelope they sealed themselves. This ensured that classification officers would not know who had completed a survey or anything about an individual's responses. A potential participant also had the option of informing the officer that she did not wish to participate, in which case the officer wrote “refused” on an envelope and placed it into the locked box. The box was metal with a small opening through which envelopes could be inserted but not removed, and the keys were maintained by the research team. Envelopes were individually numbered and tracked. Prior to the initiation of the study, the research team provided officers with instructions on administering surveys and emphasized the importance of refraining from attempts to persuade women to participate. All participating officers were required by the Virginia Tech Institutional Review Board (IRB) to complete the university's Training in Human Subjects Protection course and pass the associated test. The study was approved by the Virginia Tech IRB after a full-board review with a prisoner advocate present.

### Measures

The survey instrument was adapted from the Sexual Health Survey, developed for a study on contraceptive needs among women incarcerated at five metropolitan jails in the southeastern United States^24^ and used with permission. Several additional questions were included from the Women's Detention Center Survey, which was shared by a member of the team that developed it for the Women's Reproductive Health Services Needs Assessment in the Baltimore Women's Detention Center (not yet published). The adapted instrument consisted of 30 items.

Women were asked about their age, race, ethnicity, marital status, education, sexual orientation, employment status, living situation, and use of health care providers before jail, as well as previous pregnancies and current pregnancy, menopause, and sterilization statuses. Those who had been pregnant in the past were asked how many of their pregnancies were planned and how many had resulted in abortions and in live births.

Participants were asked whether they had vaginal sex with a man in the 3 months before jail and how many male sexual partners they had during that period. Women were asked whether they used birth control in the 3 months before jail and if so, which type(s) and if not, why not.

Women were asked whether they had vaginal sex with a man in the 5 days before jail, whether they used birth control during those encounters, and if so, which type(s).

Participants were asked about their interest in learning more about birth control methods and in starting or continuing contraception during incarceration.

Participants were asked about their likelihood of having sex with a man within 6 months of release and about their desire to get pregnant within 1 year of release. Women who did not want to get pregnant were asked what they planned to do to prevent pregnancy. Those who reported that they intended to use birth control were asked which method they planned to use.

### Data analysis

Of the 415 women invited to participate, 193 (46.5%) completed surveys. Analyses focused on those at risk for pregnancy. Ninety-seven (50.3%) of the 193 respondents were pregnant, sterilized, postmenopausal, or only had sex with women and were excluded from the analyses. One woman did not provide information on menopause status, sexual activity, or birth control use and was excluded from the analyses. The final sample size at risk for pregnancy was 95 (49.2%). Analyses were completed in Stata16. Frequency statistics were analyzed. Fisher's exact tests were used to examine potential racial and ethnic differences in contraceptive use before jail, and chi-square analyses were used to examine the association between birth control use and health care provider status before jail.

## Results

### Demographic characteristics

Demographic information is displayed in Table 1. Among the 94 women included in analyses who responded to demographic questions, ages ranged from 18 to 48 years (*M* = 28.4, SD = 6.2), with the majority White (*n* = 80, 85.1%), not Hispanic or Latina (*n* = 85, 95.5%^a^), not married (*n* = 67, 71.3%), and having at least completed high school or the General Educational Development (GED) test (*n* = 77, 81.9%).

**Table 1.** Demographic Characteristics

	*n*	%
Age group, years		
18–24	23	24.5
25–29	35	37.2
30–35	22	23.4
36 and older	14	14.9
Race		
White	80	85.1
Black or African American	9	9.6
More than one race	5	5.3
Ethnicity		
Hispanic or Latina	4	4.5
Not Hispanic or Latina	85	95.5
Marital status		
Single, not divorced or widowed	53	56.4
Married	27	28.7
Divorced or widowed	14	13.8
Highest level of education		
Less than high school	17	18.1
High school/GED	64	68.1
Technical/Associate's	9	9.6
Bachelor's degree or higher	4	4.3
Sexual orientation		
Sex with men only	76	80.1
Sex with both men and women	18	19.1
Employed (full- or part-time) before jail	34	36.6
Living situation before jail		
Spouse/sexual partner	26	28.6
Parents or other family	22	24.2
Spouse/sexual partner and child	19	20.9
Homeless	8	8.8
Children only	5	5.5
Friend(s)/Roommate(s)	5	5.5
Other	5	5.5
Alone	1	1.1
Did not have a health care provider before jail	41	43.6

Not all participants provided complete demographic information. Percentages displayed exclude missing data from the calculations.

GED, General Educational Development.

### Pregnancy history

Eighty-three women (87.4%) indicated that they had been pregnant in the past. The number of prior pregnancies reported ranged from 1 to 8 (*M* = 3.13, SD = 1.77), totaling 260 across the sample. Twenty-two (8.5%) of these were terminated through elective abortion. The majority (*n* = 182, 70.0%) of reported pregnancies resulted in live births. Seventy-eight women who reported at least one prior pregnancy responded to a question about how many were planned. Fifty-eight (74.4%) of these women indicated that they had experienced at least 1 unintended pregnancy, with 143 (57.9%) of their 247 pregnancies not reported to be planned or on purpose.

### Sexual activity and birth control use in the 3 months prior to jail

Eighty-nine (93.7%) of the 95 women included in the analyses reported that they had vaginal sex with a man at least once in the 3 months before jail. The average number of male partners during that period was 1.88 (SD = 1.78; range: 1–13).

Eighty-eight women provided information on birth control use during the 3 months before jail. The majority (*n* = 53, 60.2%) reported using contraception at least once during that period. Types of birth control women used are displayed in Table 2. The most common methods reported were male condoms (*n* = 26, 49.1%) and withdrawal (*n* = 15, 28.3%).

**Table 2.** Contraceptive Methods Women Reported using Before Jail

	3 Months before jail (*n* = 53), *n* (%)	5 Days before jail (*n* = 24), *n* (%)
Male condom	26 (49.1)	7 (29.2)
Withdrawal	15 (28.3)	3 (12.5)
IUD	11 (20.8)	6 (25.0)
Birth control pill	8 (15.1)	2 (8.3)
Contraceptive injection	7 (13.2)	4 (16.7)
Implant	5 (9.4)	3 (12.5)
Rhythm method	3 (5.7)	0 (0.0)
Contraceptive vaginal ring	1 (1.9)	1 (4.2)
Vasectomy of male partner	1 (1.9)	1 (4.2)
Female condom	1 (1.9)	0 (0.0)
Contraceptive patch	1 (1.9)	0 (0.0)

Women could select multiple types of birth control. Percentages displayed are out of the sample of women who reported using a birth control method at least once during vaginal sex with a man in the specified period. Methods not selected by any participant are omitted.

IUD, intrauterine device.

Thirty-five (39.8%) of the 88 women who had vaginal sex in the 3 months before jail and provided information about contraceptive use reported that they used no birth control during that time. Sixteen (45.7%) of these reported that they wanted to become pregnant, 11 (31.4%) reported that they did not want to become pregnant, 4 (11.4%) were unsure whether they wanted to become pregnant, and 4 (11.4%) “did not care one way or the other.” Reasons for not using birth control are displayed in Table 3. Besides wanting to become pregnant, the top reason cited for not using birth control was not thinking there was a good chance of getting pregnant (*n* = 13, 37.1%).

**Table 3.** Reasons for not using Birth Control during Vaginal Intercourse in the 3 Months before Jail

	*n* (%)
Trying to get pregnant	16 (45.7)
Did not think there was a good chance of getting pregnant	13 (37.1)
Concerned with side effects	4 (11.4)
Other	4 (11.4)
Concerned with negative health effects	3 (8.6)
Did not have sex very often	3 (8.6)
Difficult to access	2 (5.7)
Religion does not approve of birth control	1 (2.9)
Spouse/partner does not approve of birth control	1 (2.9)
Cost	1 (2.9)

Table includes data from women who reported having vaginal sex with a man and not using any contraception in the 3 months before jail (*n* = 35). Women could select multiple reasons for not using birth control. Percentages displayed are out of the sample of 35 women whose data are included. Reasons that were not selected by any participant are omitted.

Among women reporting vaginal sex during the 3 months prior to incarceration, non-White and Hispanic women had slightly higher odds of not using birth control compared with White, non-Hispanic women, but the difference was small and not significant (OR = 1.19, 95% CI [0.34–4.08], *p* = 0.78).

There was a significant association between birth control use during the 3 months prior to jail and health care provider status for those women who had vaginal sex with a man during that period, χ^2^ (1, *N* = 87) = 7.31, *p* = 0.007, such that women who reported having a health care provider were more likely to have used birth control (OR = 3.38, 95% CI [1.26–9.14]).

Eighty-one women responded to questions regarding the method they used most often and how often they used it during the 3 months before jail. A minority (*n* = 30, 37.0%) reported using a method “almost all of the time.” Table 4 summarizes method frequency results for the 61 women who did not want to get pregnant in the 3 months before jail. A minority (*n* = 28, 45.9%) of these women reported using a method “almost all of the time,” and the most common method reported was the male condom (*n* = 18, 29.5%).

**Table 4.** Contraceptive Method Used Most Often and Frequency of use in the 3 Months before Jail among Women not Wanting to Become Pregnant

	Total, *n* (%)	Almost all of the time, *n* (%)	Sometimes, *n* (%)	Rarely/Almost never, *n* (%)
No method	19 (31.1)	n/a	n/a	n/a
Male condom	12 (19.7)	5 (41.7)	4 (33.3)	3 (25.0)
IUD	6 (9.8)	6 (100)	—	—
Withdrawal	5 (8.2)	2 (40.0)	3 (60.0)	—
Implant	5 (8.2)	5 (100)	—	—
Birth control pill	3 (4.9)	2 (66.7)	—	1 (33.3)
Contraceptive injection	3 (4.9)	2 (66.7)	—	1 (33.3)
Contraceptive vaginal ring	1 (1.6)	1 (100)	—	—
Condom, withdrawal	1 (1.6)	—	1 (100)	—
Condom, pill	1 (1.6)	1 (100)	—	
Condom, injection	1 (1.6)	1 (100)	—	—
Condom, IUD	1 (1.6)	1 (100)	—	—
Condom, withdrawal, rhythm method	1 (1.6)	1 (100)	—	—
Condom, pill, injection	1 (1.6)	—	1 (100)	—
Other (partner vasectomy)	1 (1.6)	1 (100)	—	—

Table includes data from women who reported vaginal sex with a man in the 3 months before jail, did not want to become pregnant during that time, and provided information on the frequency of method use (*n* = 61). Percentages for specific methods under “Total” are out of the sample of 61 women whose data are included. Percentages for frequencies of method use are out of the women who reported using that method most often. Six participants selected more than one method from the list despite the question instructing “Choose only ONE.”

### Sexual activity and birth control use in the 5 days prior to jail

**FIG. 1.**
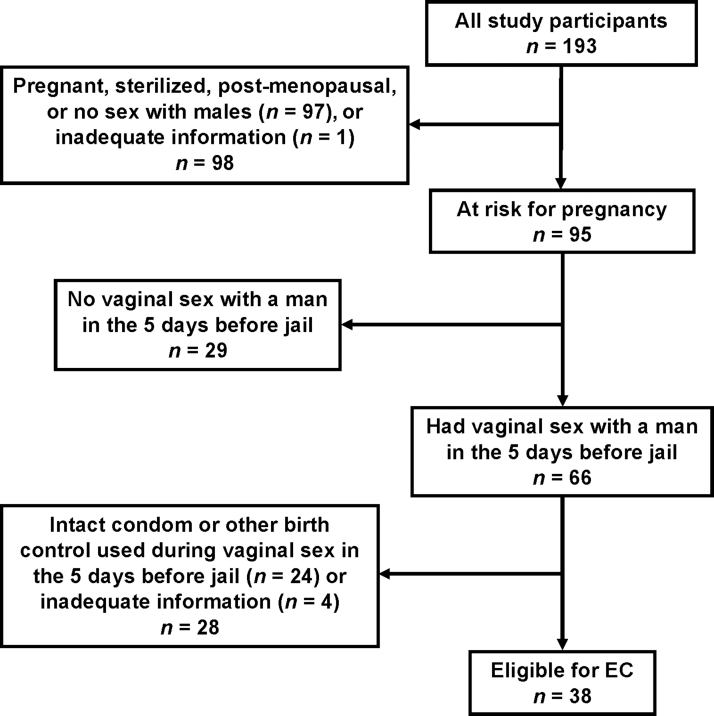
Eligibility for EC among women newly incarcerated at New River Valley Regional Jail, 2018–2019. EC, emergency contraception.

Sixty-six (69.5%) of the 95 women included in the analyses reported vaginal sex with a man at least once in the 5 days before jail. Of those, 38 (57.6%) did not use birth control and were therefore eligible for EC (Fig. 1). Twenty-four women (36.4%) reported using birth control. Three women (4.5%) did not respond to the question about whether they used birth control, and one woman (1.5%) selected “declines to answer.” Types of birth control reported using during vaginal sex in the 5 days before jail are displayed in Table 2. The most common methods reported were male condoms (*n* = 7, 29.2%) and the intrauterine device (IUD) (*n* = 6, 25.0%).

Non-White and Hispanic women had slightly higher odds of reporting unprotected vaginal intercourse in the 5 days before jail compared with White, non-Hispanic women, but the difference was small and not significant (OR = 1.07, 95% CI [0.23–5.68], *p* = 1.00).

There was a significant association between birth control use during vaginal sex in the 5 days before jail and health care provider status, χ^2^ (1, *N* = 61) = 7.88, *p* = 0.005, such that women with a provider were more likely to have used contraception (OR = 4.86, 95% CI [1.38–18.32]).

### Interest in contraceptive education and access in jail

Thirty-six (37.9%) of the 95 women included in the analyses indicated that they would be interested in learning more about birth control methods while in jail. Forty-five (47.4%) indicated that they would be interested in starting or continuing a birth control method while in jail.

### Future plans after release from jail

Most (*n* = 74, 77.9%) of the women included in the analyses reported that they were very likely or extremely likely to have sex with a man within 6 months of leaving jail. Thirty-five (36.8%) indicated that they wanted to become pregnant within 1 year of leaving jail. Of the women who said they did not want to become pregnant (*n* = 36, 37.9%) or were unsure whether they wanted to become pregnant (*n* = 24, 25.3%), 13 (21.7%) were unsure how they would prevent pregnancy, 4 (6.7%) reported that they would refrain from sex with a man, 4 (6.7%) did not provide an answer, and 2 (3.3%) selected “declines to answer.” Most (*n* = 37, 61.7%) reported that they planned to use birth control after release from jail. The most common methods that women reported they would use were male condoms (*n* = 13, 35.1%) and birth control pills (*n* = 12, 32.4%).

## Discussion

Among women who provided intention information about past pregnancies, almost three quarters indicated that they had experienced at least one unintended pregnancy. Of the 247 reported pregnancies on which intention information was provided, more than half (58%) were not planned or on purpose. By contrast, about 45% of pregnancies among women in the United States are unintended.^25^ Together with results from previous research on incarcerated women,^19^ our work suggests that unintended pregnancies are far more common among justice-involved women than among women in the general population.

Among women who did not want to become pregnant and provided information on frequency of contraceptive use, fewer than half reported using a method consistently (“almost all of the time”). Among women who reported using some form of birth control at least once in the 3 months prior to incarceration, the most common methods were male condoms and withdrawal, which have typical-use failure rates of 18% and 22%, respectively.^26^ These findings and the high prevalence of past unintended pregnancies among women in the sample suggest a high risk for future unintended pregnancies and a need for education about—and access to—more effective methods, including IUDs and implants, which have typical-use failure rates of <1% and are not user dependent.^26^ Sixteen (30.2%) of the 53 women who reported using some form of birth control in the 3 months before jail used an IUD or implant (Table 2), suggesting that these methods are acceptable to women in the study population.

Women who reported having a health care provider were more than three times more likely to have used contraception during the 3 months before jail—and nearly five times more likely to have used it during vaginal intercourse in the 5 days before jail—than women who did not have a health care provider. This suggests that increasing access to health care services for women in this population may help to prevent unintended pregnancies among them. One study reported that women incarcerated at a metropolitan correctional facility in Rhode Island were more than 20 times more likely to initiate birth control within 4 weeks of release when it was offered prerelease versus postincarceration after adjusting for sample differences,^27^ illustrating the importance of offering family planning services to incarcerated women during confinement. Rural and Appalachian women have far less access to health care than their urban, metropolitan, and non-Appalachian counterparts,^15,28^ making jail-based contraceptive access especially relevant in the context of the current study. It is imperative that interventions for this population be noncoercive and culturally sensitive. Distrust of health professionals is common among rural Appalachian women, with researchers emphasizing quality, patient-centered services provided by culturally competent individuals as key to reaching this population.^29,30^

Sixty-eight percent of the women who did not want to become pregnant and did not use birth control in the 3 months before jail reported that they did not use it because they thought it was unlikely they would become pregnant. This is consistent with results of research on women who are not incarcerated: Low perceived susceptibility to pregnancy is common among women experiencing unintended pregnancy and a strong predictor of unprotected intercourse.^31–34^ Our results suggest that educating incarcerated women to correct pregnancy risk misconceptions may prove to be a useful strategy for reducing unintended pregnancies among them and suggests a potential area for future exploration.

Thirty-eight (40.0%) of the 95 participants at risk for pregnancy reported unprotected vaginal sex within 5 days of incarceration and were, therefore, eligible for EC, which is neither available at NRVRJ nor at most correctional facilities in the United States.^11^ Results suggest that about 130 of the ∼660 women incarcerated annually at NRVRJ would be eligible for EC and could potentially benefit from its availability.^b^

The risk of pregnancy from a single random act of unprotected sex was estimated in 2001 to be 3.1%.^35^ Subsequent reevaluation of that work concluded that the risk of pregnancy from unprotected sex is actually >3.1% per event,^36^ since women are more likely to engage in intercourse near ovulation when pregnancy risk is highest.^37^ A single dose of ulipristal acetate (UPA) EC taken within 5 days of unprotected sex decreases the risk of pregnancy from that sexual activity to 1.4%.^38^ Availability of UPA EC at NRVRJ could, thus, potentially reduce the average number of pregnancies expected to occur annually among EC-eligible women at the jail from >4 to fewer than 2 if all the women to whom it was offered accepted it.

Findings suggest that about 325 of the 660 women incarcerated per year at NRVRJ could potentially benefit from prerelease access to birth control based on being at risk for pregnancy.^c^ Only 37% of women included in the analyses reported that they wanted to become pregnant within 1 year of release. More than 20% of those who did not were unsure about what they would do to prevent pregnancy. Among those who reported that they planned to use a birth control method after release, most cited male condoms or birth control pills as the options they intended to use. Both are user-dependent methods with typical-use failure rates more than 10 times higher than those of IUDs and implants.^26^ Nearly half of the respondents at risk for pregnancy indicated that they would be interested in initiating or continuing contraception while in jail. Taken together, these results support offering contraceptive education and highly effective birth control options to incarcerated women before release to prevent unintended pregnancies.

Several limitations should be addressed. First, the study relied on self-reported data potentially subject to recall bias or misreport. We were not permitted to collect information on women invited to complete a study who declined and therefore lack data on non-participants. The overall participation rate was slightly <50%, perhaps due, at least in part, to the fact that surveys were distributed by correctional staff who may have been perceived negatively by potential participants. Non-English speakers and women excluded from the standard classification process were also excluded. We, therefore, cannot be confident that the study did not suffer from selection bias or that results are generalizable to the population of women incarcerated at NRVRJ. However, our results are similar to those previously reported by researchers studying the contraceptive needs of incarcerated women and demonstrate a clear need for family planning services among many of the women at this jail. Additional research on contraceptive needs among women incarcerated at other rural Appalachian jails is needed to determine whether the results reported here are generalizable beyond NRVRJ.

## Conclusions

This study represents an important addition to existing literature in that it focused specifically on women incarcerated in rural Appalachia. Our findings add to a growing body of research supporting the need to provide access to EC on incarceration and to effective contraception before release. Incarcerated women may benefit from sexual and reproductive health education to increase their awareness of the risk of pregnancy from unprotected sex and inform them about highly effective birth control methods. Increasing access to quality sexual and reproductive health services by providing services during incarceration represents an important step in addressing some of the reproductive health disparities faced by justice-involved women.

## Data Availability

Raw data collected and analyzed for this study are available on reasonable request from the corresponding author, S.G.W., subject to a data sharing agreement. The authors have elected to refrain from publishing raw data collected for this research due to concern that doing so may compromise participants' privacy.

## Abbreviations Used

CI: confidence interval
EC: emergency contraception
GED: General Educational Development
IRB: Institutional Review Board
IUD: intrauterine device
NRVRJ: New River Valley Regional Jail
OR: odds ratio
SD: standard deviation
UPA: ulipristal acetate

## Notes

^a^ Percentage is out of the 89 women who responded to this question.

^b^ If the study sample is representative of the population of women incarcerated at NRVRJ, ∼130 of the 660 women classified per year would be expected to be eligible for EC, given that 38 (19.7%) of the 193 total respondents were eligible for EC in this study.

^c^ If the study sample is representative of the population of women incarcerated at NRVRJ, ∼325 of the 660 women classified per year would be expected to be at risk for pregnancy, given that 95 (49.2%) of the 193 total respondents were at risk for pregnancy in this study.
